# Social Interaction With Relapsed Partner Facilitates Cocaine Relapse in Rats

**DOI:** 10.3389/fphar.2021.750397

**Published:** 2021-10-04

**Authors:** Shiqiu Meng, Wei Yan, Xiaoxing Liu, Yimiao Gong, Shanshan Tian, Ping Wu, Yan Sun, Jie Shi, Lin Lu, Kai Yuan, Yanxue Xue

**Affiliations:** ^1^ National Institute on Drug Dependence and Beijing Key Laboratory of Drug Dependence, Peking University, Beijing, China; ^2^ NHC Key Laboratory of Mental Health (Peking University), National Clinical Research Center for Mental Disorders (Peking University Sixth Hospital), Peking University Sixth Hospital, Peking University Institute of Mental Health, Beijing, China; ^3^ Chinese Institute for Brain Research, Beijing, China

**Keywords:** social interaction, cocaine, relapse, peer influence, addiction, animal model

## Abstract

Social factors strongly contribute to drug use and relapse, and epidemiological studies have found that members of peer groups influence each other to use drugs. However, previous animal models mostly failed to incorporate social factors and demonstrate the effects of social partners on drug addiction and relapse. In the present study, we investigated the transfer of relapse to cocaine seeking between drug-addicted partners in rats. Male Sprague–Dawley rats were pair-housed and subjected to training and extinction of cocaine self-administration and conditioned place preference (CPP). 24 h after extinction test, the targeted rats interacted with a cocaine-primed (relapsed) partner or stranger, or saline-injected (unrelapsed) partner for 30 min, after which the targeted rats were tested for drug seeking behavior. We found that social interaction with a relapsed partner increased drug seeking behavior in cocaine self-administration and CPP models in rats, while social interaction with an unrelapsed partner or relapsed stranger had no effect on cocaine seeking. Moreover, the effect of social interaction on cocaine seeking could last for at least 1 day. Our findings demonstrate a facilitation effect of relapsed social partners on drug relapse in rats and provide a novel animal model for social transfer of drug relapse.

## Introduction

Drug addiction is a chronic recurrent disease bringing heavy burden to individuals, families, and society (Volkow and Boyle, 2018), and is characterized by high rates of relapse even after treatment (Sinha, 2011). Social and environmental factors are acknowledged as determinants of drug use and relapse, and among various factors, social partners may be critical (Bahr et al., 2005; Simons-Morton and Chen, 2006). Epidemiological investigations have demonstrated that people easily become addicts if their friends are addicted to drugs (Walden et al., 2004; Bahr et al., 2005; Simons-Morton and Chen, 2006). Some clinical research indicates that social interaction with alcohol-addicted partners not only accelerated acquisition of addiction, but also enhanced alcohol tolerance (Caudill and Marlatt, 1975; Lied and Marlatt, 1979; Caudill and Kong, 2001; Larsen et al., 2010; Kirkpatrick and de Wit, 2013), demonstrating that social interaction with addicted partners may play a facilitation effect on addiction. Having drug-using friends has also been found to increase risks of heroin relapse in patients under methadone maintenance treatment (Li et al., 2012). On the contrary, joining recovery communities like Alcoholics Anonymous (Kelly et al., 2012; Frings et al., 2021) and interacting with non-addicted peers to change their social network (Bathish et al., 2017) promotes recovery from addiction. However, due to ethical reasons, human studies on impact of social partners on illicit drug addiction are limited.

Animal models are critical to reveal the neural mechanisms of drug addiction and help clinicians to develop potential treatments, whereas only a few studies have incorporated social factors and demonstrated the effects of social partners on drug addiction. For example, Smith et al. (2015) have found that self-administration behavior of rats is promoted when they are reared with a partner and both trained with cocaine self-administration, while the addictive behavior decreases when the partners have no access to cocaine. Compared with peers who did not experience cocaine self-administration training, rats showed more preference to partners with whom they were trained for self-administration together (Smith and Pitts, 2014), especially those who took similar drugs (Smith et al., 2015). Similar results were also found in the conditioned place preference (CPP) model (Larsen et al., 2010; Koordeman et al., 2011). The above findings suggest that social interaction with addicted partners affects the acquisition and maintenance of addiction. However, the impact of social partners on addiction relapse needs further study.

Numerous studies have demonstrated that after abstinence or extinction, drug craving can still be triggered once the animals are exposed to drug or drug-related cues (e.g., sound and light) (Davachi, 2006; Brandon et al., 2007; Goldfarb and Sinha, 2018). Thus, we speculate that drug craving may also be prompted by social interaction with relapsed partners. In the present study, we established an animal model of social transfer of relapse and investigated the effect of social interaction on cocaine seeking behavior after extinction.

## Materials and Methods

### Experimental Design

Experiment 1 Effects of social interaction with relapsed partner. Rats were pair-housed and subjected to 10 days cocaine self-administration training, followed by extinction. 24 h after extinction test, one of the two rats (relapsed partner) was primed with cocaine (10 mg/kg, i.p.), and subjected to 1 h reinstatement test. Next, the relapsed partner was put back to their homecage, and interacted with the other rat (targeted rat) for 30 min. Then the targeted rat was immediately subjected to 1 h reinstatement test.

Experiment 2 Effects of social interaction with unrelapsed partner. Rats were pair-housed and subjected to cocaine self-administration training and extinction. 24 h after extinction test, one of the two rats (unrelapsed partner) was injected with saline (1 ml/kg, i.p.), and subjected to reinstatement test. Next, the unrelapsed partner was put back to their homecage, and interacted with the other rat (targeted rat) for 30 min. Then the targeted rat was subjected to reinstatement test immediately.

Experiment 3 Effects of social interaction with relapsed partner for different time. Rats were pair-housed and subjected to cocaine self-administration training and extinction. 24 h after extinction test, one of the two rats (relapsed partner) was primed with cocaine (10 mg/kg, i.p.), and subjected to reinstatement test. Next, the other rat (targeted rat) interacted with the relapsed partner in their homecage for 0, 10, 30, or 60 min, followed by reinstatement test immediately.

Experiment 4 The maintenance of facilitation effect after 30 min social interaction with the relapsed partner. Rats were pair-housed and subjected to cocaine self-administration training and extinction. 24 h after extinction test, one of the two rats (relapsed partner) was primed with cocaine (10 mg/kg, i.p.), and subjected to reinstatement test. Next, the other rat (targeted rat) interacted with the relapsed partner in their homecage for 30 min, and subjected to reinstatement test immediately (0 min), or 1–7 days later.

Experiment 5 Effects of social interaction with relapsed stranger. Rats were pair-housed and subjected to cocaine self-administration training and extinction. 24 h after extinction test, a rat kept in another cage (relapsed stranger) was injected with a single dose of cocaine (10 mg/kg, i.p.) and subjected to reinstatement test. Then the relapsed stranger was put into the homecage of the targeted rat (its partner was removed from the cage) for 30 min social interaction, and the targeted rat was subjected to reinstatement test immediately.

Experiment 6 Effects of social interaction on relapse to cocaine-induced CPP. Rats were pair-housed and subjected to the baseline test. After CPP training and extinction, the rats were subjected to the extinction test. 24 h later, the targeted rats interacted with relapsed partners, unrelapsed partners, or relapsed strangers for 30 min in homecages, and then were subjected to reinstatement test immediately.

### Subjects

We used adult male Sprague–Dawley (SD) rats (280–300 g upon arrival) purchased from Vital River Laboratories. The rats were pair-housed in an animal facility with appropriate temperature (22 ± 2°C) and humidity (50 ± 10%), as well as freely accessible water and food. The lighting time was controlled, under a 12 h light/dark circle. All behavioral experiments were performed under the dark circle and in accordance with the National Institutes of Health Guide for the Care and Use of Laboratory Animals, and approved by the Biomedical Ethics Committee for Animal Use and Protection of Peking University.

### Surgery

Rats were under anesthetic through pentobarbital sodium (20 mg/ml, 60 mg/kg, i.p.) and received catheters insertion into the right jugular vein with the tip reaching the opening of right atrium. After the surgery, rats were recovered for 7 days with penicillin administration (resolved in 0.2% heparin sodium) every day, preventing infection and cannula blocking.

### Intravenous Cocaine Self-Administration Training

Cocaine-HCl was purchased from the Qinghai Pharmaceutical Factory and resolved in 0.9% saline (5 mg/ml).

The training procedure was based on our previous studies (Xue et al., 2012; Luo et al., 2015). The chambers (AniLab Software and Instruments) were equipped with two nosepoke holes 9 cm above the underside, one was active-nosepoke hole and the other was inactive-nosepoke hole. The two rats in each cage were trained at the same time in different chambers for fixed ratio 1 (FR1) cocaine self-administration training for 10 days during three 1 h sessions per day with 5 min intervals. Every session started from the illumination of a house light. Poking to the active-nosepoke hole led to a cocaine infusion (0.75 mg/kg), accompanied with a 5 s tone-light cue, while poking to the inactive-nosepoke hole did not result in cocaine infusion or tone-light cue. There was a 40 s time-out phase between each infusion, after which the house light would turn on again. The number and time of active nosepokes, inactive nosepokes, and infusions were recorded. To prevent rats administrating overdose of cocaine, the number of infusions was limited to 20 times in each session. After training, the rats were returned to their homecages.

### Extinction of Self-Administration

The two rats in each cage were subjected to extinction at the same time in different chambers. During the extinction sessions, the conditions were the same as those during the self-administration training. But there was no cocaine infusion after rats poked the active-nosepoke hole. At the end of extinction every day, they were put back in their homecages. The extinction was performed until the number of active-nosepokes decreased to below 20% of the mean nosepokes during the last 3 days of self-administration training for at least two consecutive days. 24 h later, the rats were subjected to test for drug seeking (extinction test).

### Training of Cocaine-Induced CPP

The procedure was based on our previous studies (Xue et al., 2012). Three-chamber apparatuses were used, and time the rats spent in each chamber was recorded. The two rats in each cage were trained at the same time in different apparatuses. For the baseline test (day 1), there were no partitions among the chambers. Rats were put into the middle chamber and allowed to move freely for 15 min. Rats that presented a preference for one of the boxes (resistance time >540 s) were ruled out. Then the rats were trained for cocaine-induced CPP for 8 days. Rats received intraperitoneal cocaine (10 mg/kg, day 2/4/6/8) or saline (1 ml/kg, day 3/5/7/9) injections alternatively and were confined to the conditioning chambers for 45 min after injection. The rats were returned to their homecages after training every day. 24 h after the last-day training (day 10), drug seeking test (training test) was performed.

### Extinction of Cocaine-Induced CPP

The two rats in each cage were subjected to extinction at the same time in different chambers. The conditions for extinction were the same as training except that no injections were given. At the end of extinction every day, they were put back in their homecages. After 8 days of extinction, rats were subjected to drug seeking test (extinction test).

### Drug Priming

For cocaine priming or saline injection, the rats were intraperitoneally injected with 10 mg/kg cocaine or 1 ml/kg saline, and then they were delivered to drug seeking test (reinstatement test).

### Social Interaction

To investigate the effect of social interaction with a relapsed partner, one rat in each cage was intraperitoneally injected with cocaine (10 mg/kg), and tested for drug seeking. Next, it was put back to its homecage as a relapsed partner, and interacted with the other rat (targeted rat) for 0, 10, 30, or 60 min.

To test the effect of social interaction with an unrelapsed partner, saline (1 ml/kg) was intraperitoneally injected to one rat in each cage, and then drug seeking was tested. Then it was put back to its homecage as an unrelapsed partner, and interacted with the other rat (targeted rat) for 30 min.

To investigate the effect of social interaction with a relapsed stranger, one rat in each cage was intraperitoneally injected with cocaine (10 mg/kg) and tested for drug seeking. Then it was put back to another cage as a relapsed stranger, and interacted with the rat (targeted rat) in this cage for 30 min. The partner of the targeted rat was removed from their homecage in advance.

After social interaction, the targeted rats were subjected to drug seeking test (reinstatement test).

### Drug Seeking Test

The conditions during the drug seeking tests in the self-administration model were the same as those during the extinction sessions, and the tests lasted for 1 h.

The conditions during the drug seeking tests in the CPP model were the same as those during the baseline test, and the tests lasted for 15 min. The time spent in the cocaine-paired chamber minus the time spent in the saline-paired chamber was calculated as the CPP score.

### Statistical Analysis

All of the statistical analyses were performed using SPSS 20.0 software (SPSS, Chicago, IL, United States). The data were expressed as mean ± SEM, and analyzed by repeated measures analysis of variance (ANOVA) with appropriate within-group factors for each experiment (see Results), followed by least significant difference (LSD) post hoc tests in Experiment 6. Values of *p* < 0.05 were considered statistically significant.

## Results

### Social Interaction With Relapsed Partner Triggered Relapse to Cocaine Seeking

**FIGURE 1:**
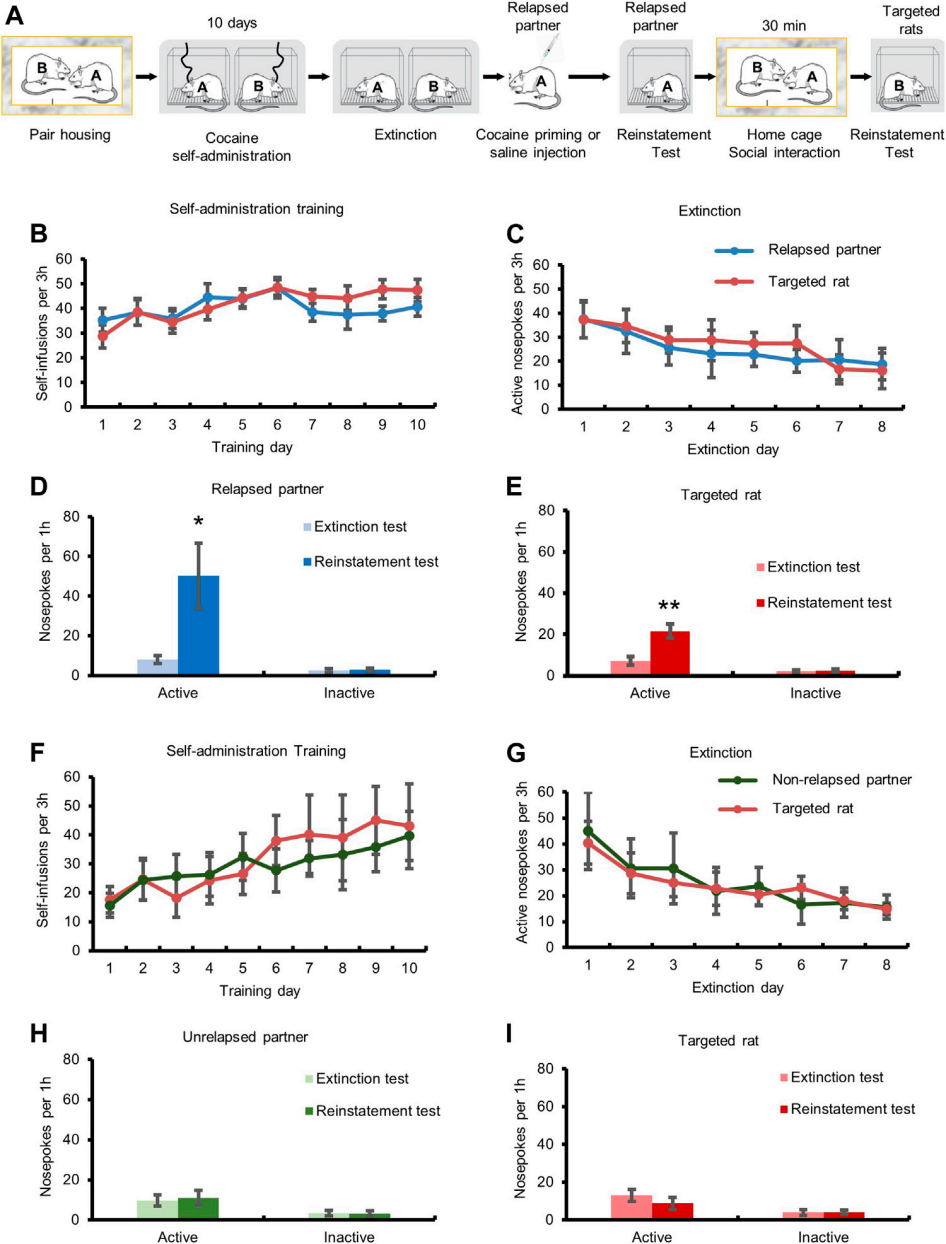
Social interaction with relapsed partner induced relapse to cocaine seeking behavior. **(A)** Experimental timeline. **(B,F)** Targeted rats and partners acquired cocaine self-administration training. **(C,G)** Drug-seeking behavior of targeted rats and partners was extinguished. **(D)** Cocaine injection triggered drug seeking behavior. **(E)** Drug-seeking behavior of targeted rats was increased after social interaction with relapsed partner (Data are shown as mean ± SEM. **p < 0.01, compared with extinction test. *n* = 11). **(H)** Saline injection had no effect on drug seeking behavior. **(I)** Drug-seeking behavior of targeted rats had no change after social interaction with unrelapsed partner (Data are shown as mean ± SEM. *n* = 7).

First, we investigated the effect of social interaction on cocaine seeking behavior in the self-administration model. We first explored whether social interaction with a relapsed partner would induce relapse (Figures 1A–C). The repeated measures ANOVA with the within-subjects factors (extinction test and reinstatement test) showed that cocaine injection increased the number of active-nosepokes (F_1,10_ = 6.670, *p <* 0.05), and had no effect on the number of inactive-nosepokes (*p* > 0.05) in the reinstatement test, indicating that cocaine priming induced reinstatement of drug seeking (Figure 1D). In the targeted rats group, the repeated measures ANOVA of nosepokes with the within-subjects factors (extinction test and reinstatement test) revealed that the number of active-nosepokes was increased after social interaction with relapsed partner (F_1,10_ = 17.862, *p <* 0.01), and inactive-nosepokes had no change (*p* > 0.05) in the reinstatement test, suggesting that social interaction with relapsed partner resulted in relapse of cocaine seeking (Figure 1E).

We also assessed the effect of social interaction with an unrelapsed partner on cocaine seeking (Figures 1A,F,G). The repeated measures ANOVA showed that the number of active-nosepokes had no significant change (*p* > 0.05) after saline injection in the reinstatement test, indicating that saline injection did not induce cocaine relapse (Figure 1H). The repeated measures ANOVA of the number of active-nosepokes of the targeted rats showed no significant change (both *p* > 0.05) after social interaction with unrelapsed partner, indicating that social interaction with unrelapsed partner did not induce relapse of cocaine seeking.

**FIGURE 2:**
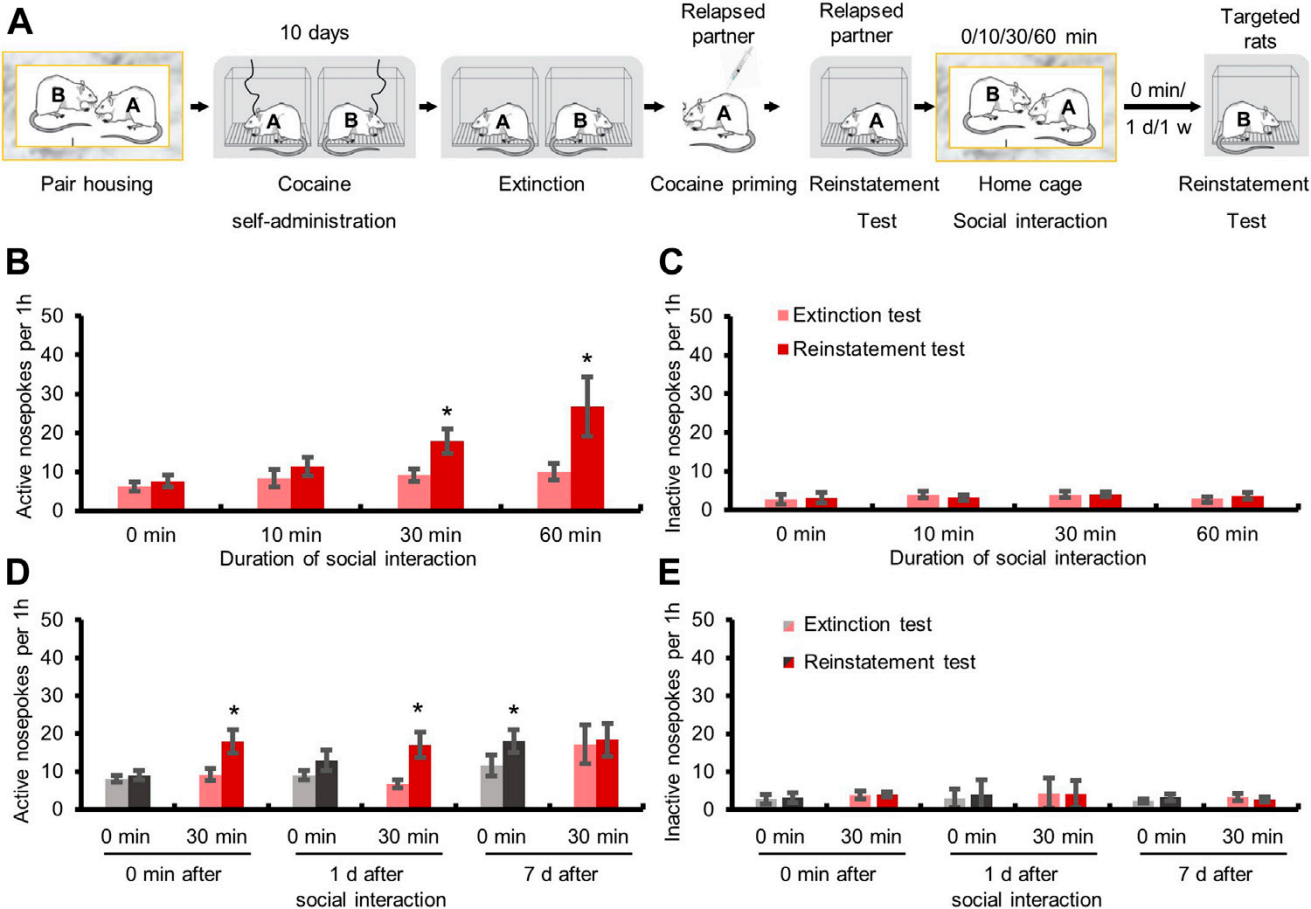
Social interaction with relapsed partners for over 30 min triggered relapse to drug-seeking behavior and this effect lasted for at least 1 day. **(A)** Experimental timeline. **(B)** Active nosepokes were enhanced after social interaction with relapsed partner for 30 or 60 min. **(C)** Inactive nosepokes had no change after social interaction (Data are shown as mean ± SEM. *p < 0.05, compared with extinction test. *n* = 7–9). **(D)** Active nosepokes were increased immediately or 1 day after 30 min social interaction with relapsed partner. **(E)** Inactive nosepokes had no change after social interaction (Data are shown as mean ± SEM. *p < 0.05, compared with extinction test. *n* = 6–9).

We next explored the effect of different interaction time on the relapse to cocaine seeking, and targeted rats interacted with relapsed partner for 0, 10, 30, or 60 min (Figure 2A). The repeated measures ANOVA showed that the numbers of active-nosepokes of the targeted rats were elevated after 30 min (F_1,7_ = 9.171, *p <* 0.05) and 60 min (F_1,6_ = 7.088, *p <* 0.05) social interaction with the relapsed partner, but not after 0 min (no social interaction) or 10 min social interaction (Figure 2B, both *p* > 0.05). The above findings indicated that social interaction required a certain amount of time (no less than 30 min) to produce the facilitation effect on relapse.

Then we further tested how long the facilitation effect of social interaction with relapsed partner on relapse could last (Figure 2A). The repeated measures ANOVA showed that the targeted rats had a higher number of active-nosepokes when tested immediately (F_1,7_ = 9.171, *p <* 0.05), or 1 day (F_1,5_ = 13.164, *p <* 0.05), but not 7 days (*p* > 0.05), after 30 min social interaction with relapsed partners, while no significant change was found when the targeted rats which did not interact with relapsed partners were tested immediately or 1 day later (both *p* > 0.05). Interestingly, when tested 7 days later, the number of active-nosepokes of the targeted rats that did not experience social interaction was increased during the drug seeking test (F_1,6_ = 6.161, *p <* 0.05), possibly resulting from spontaneous recovery of drug seeking (Figure 2D). These results demonstrated that the effect of social interaction on cocaine seeking lasted for at least 1 day.

### Social Interaction With Relapsed Strangers did not Induce Relapse to Cocaine Seeking

**FIGURE 3:**
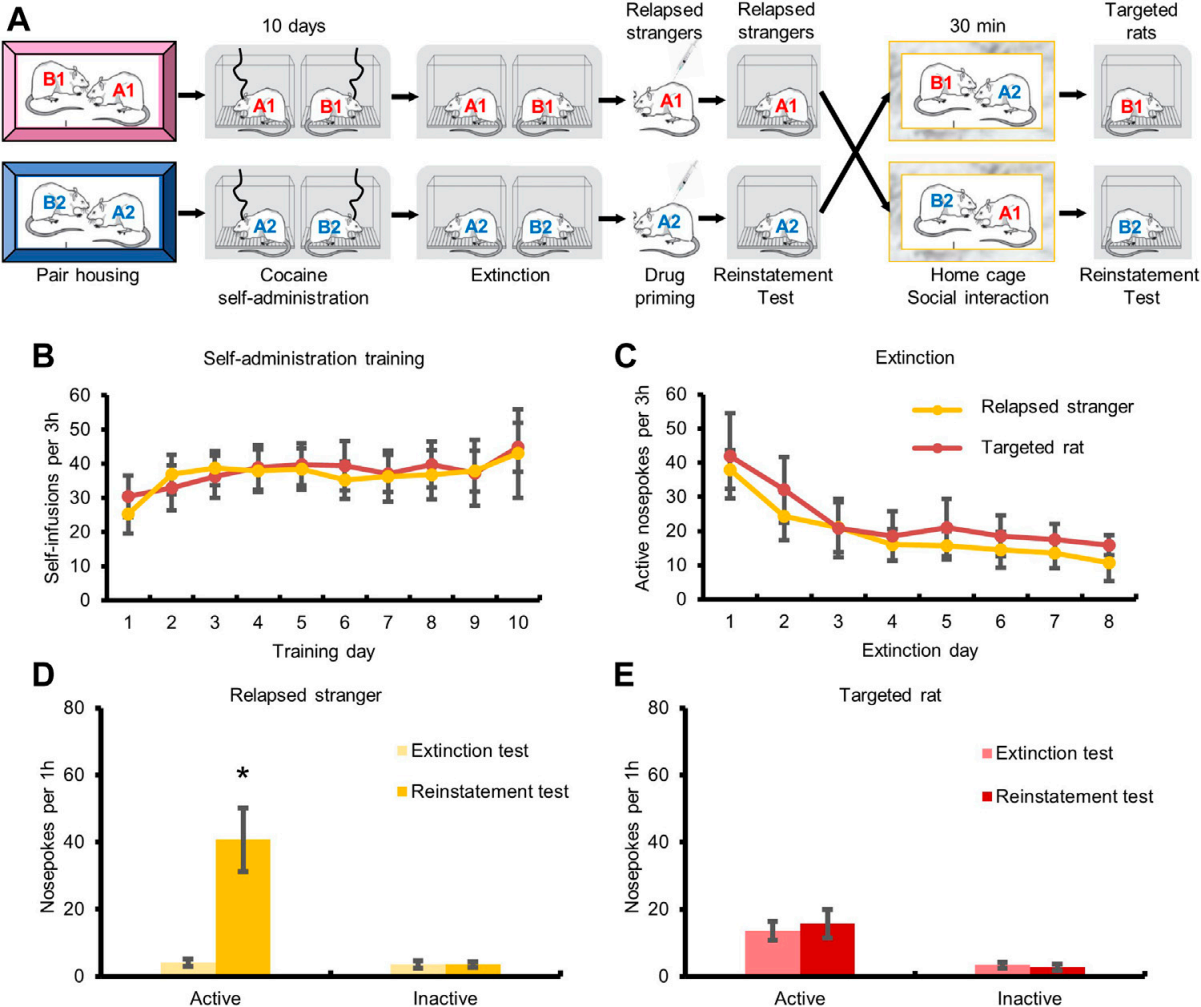
Social interaction with relapsed strangers had no effect on drug-seeking behavior. **(A)** Experimental timeline. **(B)** Targeted rats and strangers acquired cocaine self-administration training. **(C)** Drug-seeking behavior of targeted rats and strangers was extinguished. **(D)** Cocaine injection triggered drug-seeking behavior. **(E)** Drug-seeking behavior of targeted rats had no change after social interaction with relapsed stranger (Data are shown as mean ± SEM. *p < 0.05, compared with extinction test. *n* = 9).

Next, we investigated whether social interaction with relapsed strangers could also induce cocaine relapse (Figures 3A–C). The repeated measures ANOVA with within-subject factors (extinction test and reinstatement test) showed that the numbers of active-nosepokes of the strangers were elevated after cocaine injection (Figure 3D, F_1,8_ = 13.857, *p <* 0.05). No significant change was found in the numbers of active-nosepokes of the targeted rats after social interaction with relapsed strangers (Figure 3E, *p* > 0.05). The above findings indicated that transfer of cocaine relapse occurred during social interaction with relapsed partner rather than relapsed stranger.

### Social Interaction With Relapsed Partners Promoted Relapse to Cocaine-Induced CPP

**FIGURE 4:**
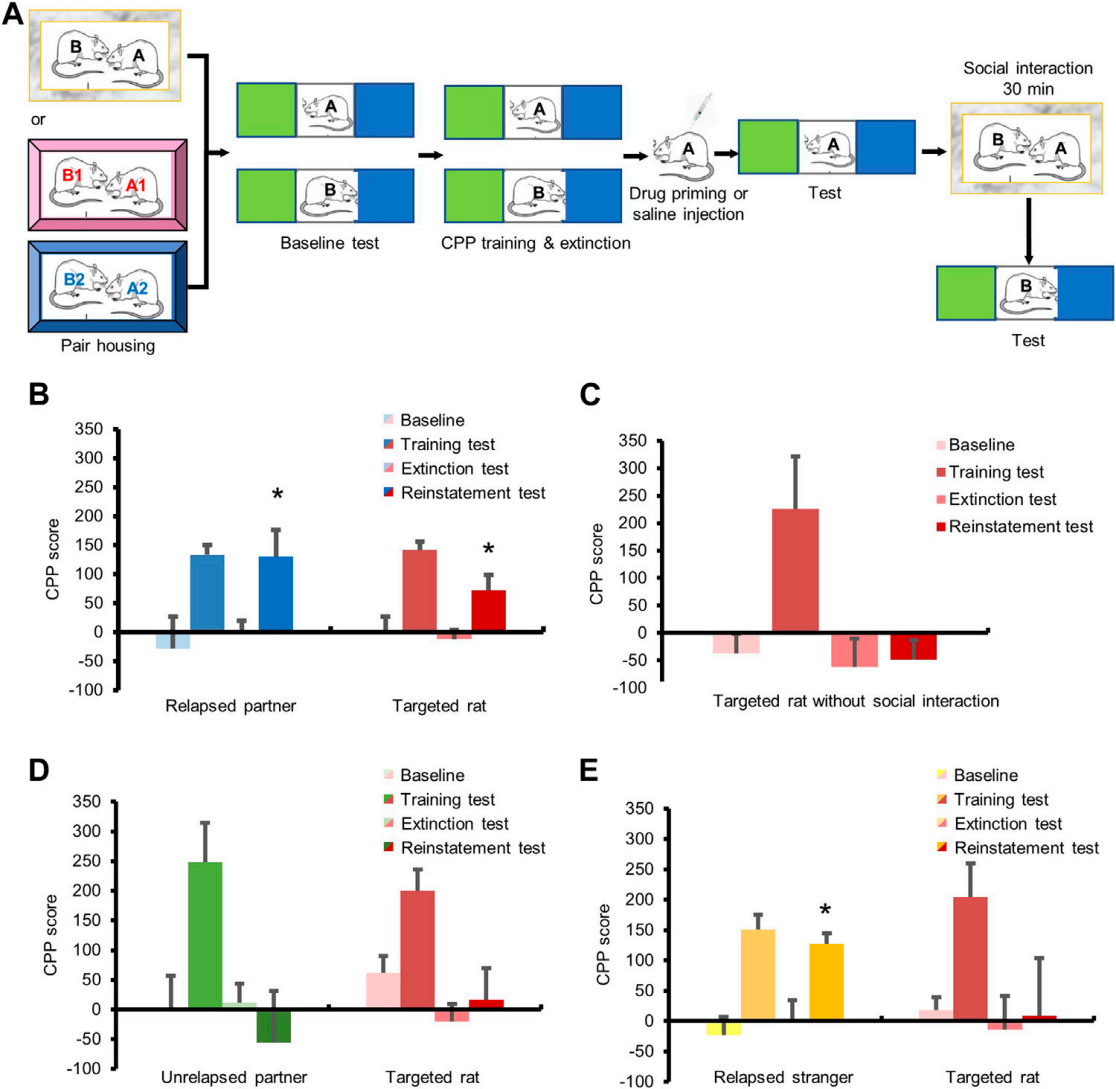
Social interaction with relapsed partners triggered relapse in cocaine CPP model. **(A)** Experimental timeline. **(B–E)** All rats obtained cocaine CPP training and extinction. **(B)** Cocaine injection triggered relapse of the partners, and social interaction with relapsed partner enhanced drug seeking of targeted rats. **(C)** No social interaction had no influence on relapse of targeted rats. **(D)** Social interaction with unrelapsed partner had no effect on relapse of targeted rats. **(E)** Social interaction with relapsed stranger had no effect on relapse of targeted rats. (Data are shown as mean ± SEM. *p < 0.05, compared with extinction test. *n* = 6–7).

Finally, we validated the facilitation effect of social interaction on cocaine relapse through the cocaine-induced conditioned place preference (CPP) model, which is also a widely used animal model to study drug addiction (Figure 4A). The repeated measures ANOVA of CPP scores of relapsed partners with within-subjects factors (baseline test, training test, extinction test, and reinstatement test), showed a significant main effect (Figure 4B, F_3,15_ = 4.665, *p <* 0.05). Post hoc tests revealed significant differences between baseline test and training test (*p <* 0.05), training test and extinction test (*p <* 0.01), or extinction test and reinstatement test (*p <* 0.05). Meanwhile, the repeated measures ANOVA of CPP scores of the targeted rats with within-subjects factors (baseline test, training test, extinction test, and reinstatement test), showed a significant main effect (Figure 4B, F_3,15_ = 14.166, *p <* 0.01). Post hoc tests revealed significant differences between baseline test and training test (*p <* 0.01), training test and extinction test (*p <* 0.01), or extinction test and reinstatement test (*p <* 0.05). The above results suggested that social interaction with relapsed partners promoted the transfer of cocaine relapse. But if the targeted rats did not interact with relapsed partners, no difference was found between the extinction test and reinstatement test (Figure 4C, *p* > 0.05).

For the unrelapsed partners, the repeated measures ANOVA of CPP scores showed a significant main effect (Figure 4D, F_3,15_ = 3.785, *p <* 0.05). Post hoc tests revealed significant differences between the baseline test and training test (*p <* 0.01), or training test and extinction test (*p <* 0.05), but no difference between the extinction test and reinstatement test (*p* > 0.05). The repeated measures ANOVA of the CPP scores of targeted rats which interacted with unrelapsed partners revealed a significant main effect (Figure 4D, F_3,15_ = 9.560, *p <* 0.01). Post hoc tests revealed significant differences between baseline test and training test (*p <* 0.05), or training test and extinction test (*p <* 0.01). No change was found between the extinction test and reinstatement test (*p* > 0.05), demonstrating that social interaction with unrelapsed partners had no effect on the transfer of cocaine relapse.

A significant main effect was shown for the CPP scores of the relapsed strangers by repeated measures ANOVA (Figure 4E, F_3,18_ = 14.296, *p <* 0.01), and post hoc tests revealed significant differences between baseline test and training test (*p <* 0.01), training test and extinction test (*p <* 0.01), or extinction test and reinstatement test (*p <* 0.05). Meanwhile, the repeated measures ANOVA of CPP scores of the targeted rats interacting with relapsed strangers showed a significant main effect (Figure 4E, F_3,18_ = 4.050, *p <* 0.05). Post hoc revealed significant differences between baseline test and training test (*p <* 0.01), or training test and extinction test (*p <* 0.05), while no change was found between the extinction test and reinstatement test (*p* > 0.05), indicating that social interaction with relapsed strangers did not affect cocaine seeking.

## Discussion

In the present study, we proposed a novel animal model to explore the effects of social partners on relapse, and investigated the social transfer of drug relapse based on two classic behavioral paradigms, cocaine self-administration and CPP. We found that social interaction with relapsed (cocaine-primed) partners for at least 30 min induced relapse to cocaine seeking behavior, and the effect lasted for over 24 h. In contrast, neither social interaction with relapsed strangers nor with unrelapsed partners had a facilitation effect on cocaine relapse.

Social experiences are important influential factors for drug addiction and relapse (Heilig et al., 2016). In previous studies, many animal models of addiction relapse have been established and validated that drug priming, drug-related cues or context, and stress can induce reinstatement/relapse after extinction (Pohorecky, 2008; Neisewander et al., 2012; Heilig et al., 2016). However, only a few models incorporated social factors, and there remain debates about the role of social factors in relapse. Ribeiro Do Couto et al. (2009) found that social isolation before CPP training or exposure to social defeat stress before cocaine priming promoted relapse to cocaine-induced CPP, whereas exposure to a non-addicted female mouse or brief social interaction with a non-addicted and non-aggressive male mouse before cocaine priming could reduce relapse (Ribeiro Do Couto et al., 2009). Venniro et al. (2018) built a model of choice between drugs and social interaction, and found that access to social interaction with non-addicted rats, as a social reward, could prevent methamphetamine self-administration and relapse. While some studies demonstrated that social interaction with addicted peers promoted the possibility of addiction (Doty and de Wit, 1995; Kirkpatrick and de Wit, 2013), other studies reported the inhibitory effects of social factors on addiction (Deatherage, 1972; Weisinger et al., 1989). For example, compared with rats kept in pairs, rats kept solely got more morphine in the social environment (Alexander et al., 1981; Raz and Berger, 2010). If these isolated rats were allowed to interact with other non-addicted peers before the test, the preference for morphine was also attenuated (Hadaway et al., 1979; Raz and Berger, 2010). Based on the findings above, we speculated that social interaction with non-addicted or unrelapsed peers may prevent relapse, while interaction with addicted or relapsed peers may facilitate relapse. We found that interaction with relapsed partner, but not unrelapsed partner, after extinction promoted cocaine seeking. The present study provides a perspective that relapsed and unrelapsed partners produce different effects on relapse. Our results and previous findings confirmed the social-learning theory (Peitz et al., 2013) which demonstrates that partners of a group affect the behavior of other members.

However, the role of social interaction on addiction and relapse is complex and the state of partners is not the only determining factor. Some research revealed that interaction with non-addicted partners produced a dose-response effect (Wolffgramm, 1990; Wolffgramm and Heyne, 1991). Compared with isolated rats, rats that interacted with members of peer group partly (there was a segregation network between them) was prevented from alcohol seeking, while rats that interacted with partners completely (there was no segregation network between them) presented increased alcohol intake (Wolffgramm, 1990), suggesting there was a dose-effect relationship between interaction degree and alcohol intake. Moreover, the gender of partners also has an effect. A study using prairie voles found that male prairie voles which were pair-housed with other male ones showed more preference to alcohol (Anacker et al., 2011), whereas alcohol preference of those kept with female prairie voles had no change (Hostetler et al., 2012). The above findings reveal that the effect of social interaction on addiction and relapse depends on not only whether the partners got addicted or relapsed but also the interaction degree and gender of partners. Thus, further studies are needed to investigate the effect of social interaction at different degrees or in different communicating ways (i.e., olfactory, auditory, visual, or tactile communication), or with peers having different genders on drug relapse.

Previous research has showed various brain regions responsible for social interaction and provides an insight into the “social brain” (Insel and Fernald, 2004). It includes the brain areas activated during the social cognition tasks, like the regions for social identification, environmental assessment, social motivation, and behavior execution. Both human and animal studies verified the critical effect of the medial prefrontal cortex (mPFC), hippocampus, amygdala, and thalamus on social interaction (Kas et al., 2014), and meanwhile, these brain areas also contribute to cocaine addiction and relapse (Brandon et al., 2007). Notably, EI Rawas et al. (2012) found that similar brain regions were activated by cocaine-induced CPP and social interaction-induced CPP, including prelimbic, infralimbic, orbitofrontal, and cingulate cortex, as well as striatum, central and basolateral amygdala, and ventral tegmental areas, which had been proved to be associated with cocaine conditioned stimuli and social interaction (Thomas et al., 2003; Salchner et al., 2004; Miller and Marshall, 2005). Inhibiting protein synthesis in the mPFC (Marcondes et al., 2020), hippocampus (Garrido Zinn et al., 2016), or amygdala (Gur et al., 2014; Garrido Zinn et al., 2016) impaired the discrimination ability between familiar peers and strangers, suggesting that social interaction with relapsed partners instead of strangers possibly activated these brain regions and retrieved seeking for cocaine. This may be why only interaction with relapsed partners instead of strangers can induce cocaine relapse. Besides, the mirror neuron system is the hub for understanding other’s emotions, intentions, and actions (Rizzolatti et al., 1999; Rizzolatti and Craighero, 2004; Molenberghs et al., 2009). Mirror neurons are widely distributed in the inferior frontal gyrus, primary somatosensory cortex, supplementary motor area, and other cortex (Molenberghs et al., 2009), and a study recruiting patients with lesions in the lateral prefrontal cortex showed deficits in understanding other’s emotion, indicating mirror neurons also exist in the prefrontal cortex (Perry et al., 2017). Therefore, mirror neurons in the prefrontal cortex may play an important role in the social transfer of drug relapse, which needs further investigation.

There are some limitations in our work. First, we established a novel model for the transfer of relapse between partners, but it remains unclear whether it is applicable in other drugs like heroin and nicotine. Furthermore, social interaction may produce similar effects in other disorders such as depression and post-traumatic stress disorder, which also needs further investigation. Second, we did not investigate the neural mechanisms underlying the effects of social interaction on relapse. Future studies need to be conducted to explore how the information is transmitted from the relapsed partners to the targeted rats, and how the brain areas mediating social information processing activate the ones required for addiction and relapse.

In conclusion, we introduced a novel animal model for the social transfer of drug relapse, and found that cocaine relapse could be induced by social interaction with relapsed partner, but not unrelapsed partner or relapsed stranger. Our findings suggest the importance of living in drug-free communities and keeping away from relapsed partners for abstinent drug users, and emphasize the necessity of paying attention to social interaction factors when formulating prevention and treatment strategies for relapse.

## Data Availability

The raw data supporting the conclusion of this article will be made available by the authors, without undue reservation.
